# Supplementary material for: DMRforPairs: identifying Differentially Methylated Regions between unique samples using array based methylation profiles
Source: BMC Bioinformatics. 2014 May 15;15:141. doi: 10.1186/1471-2105-15-141 (PMC4046028; doi:10.1186/1471-2105-15-141)
Supplement: Additional file 2 — DMRforPairs output for the comparison of A431-MCF7 and NA17018-NA17105. Please start from the HTML files in each folder. Available via the BMC Bioinformatics website. [file 1471-2105-15-141-S2.zip › 1394847754114233_MOESM2_ESM/NA17105M_NA17018F/figures/6447.pdf]

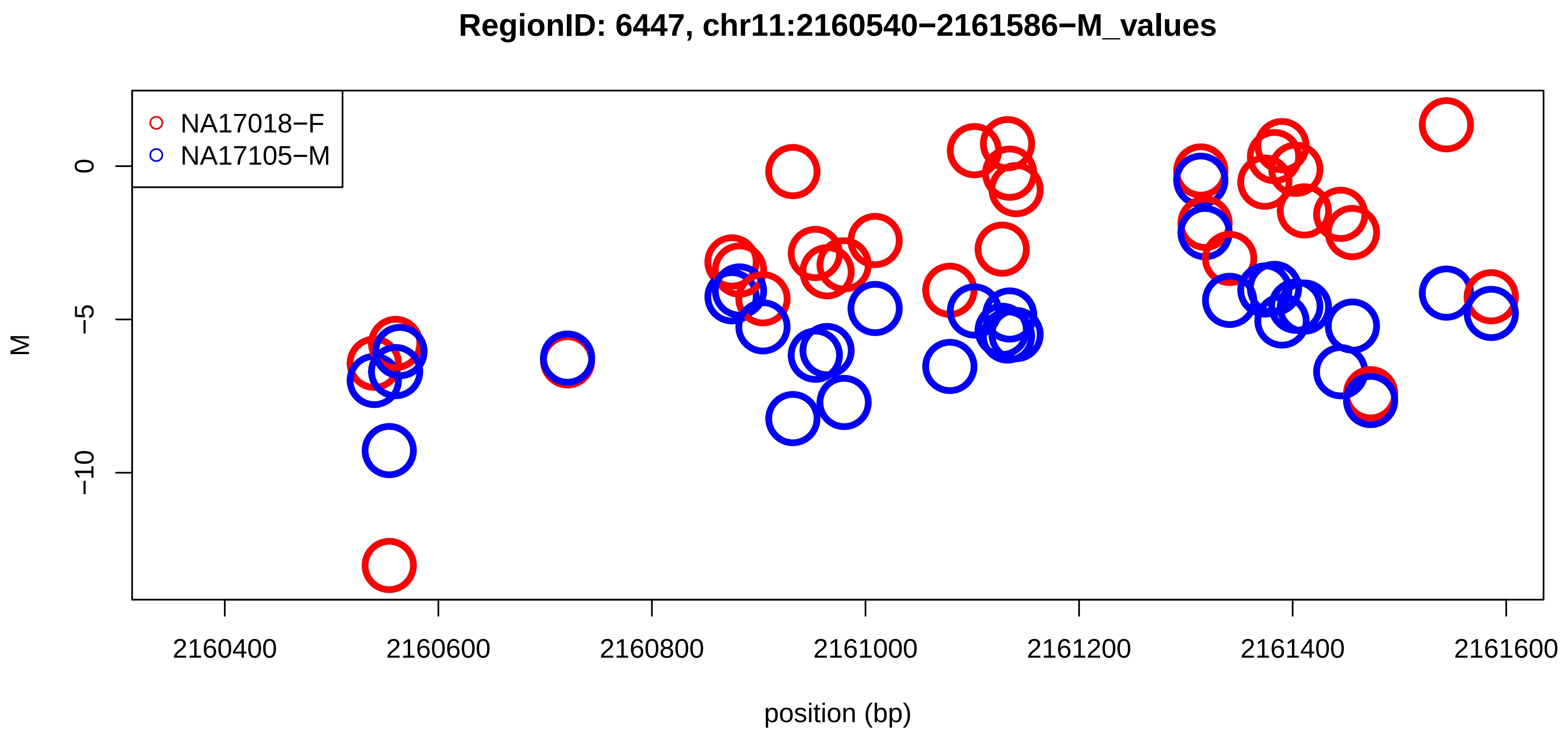

RegionID: 6447, chr11:2160540−2161586−M_values
NA17018−F
NA17105−M
0
−5
−10
M
2160400
2160600
2160800
2161000
2161200
2161400
2161600
position (bp)


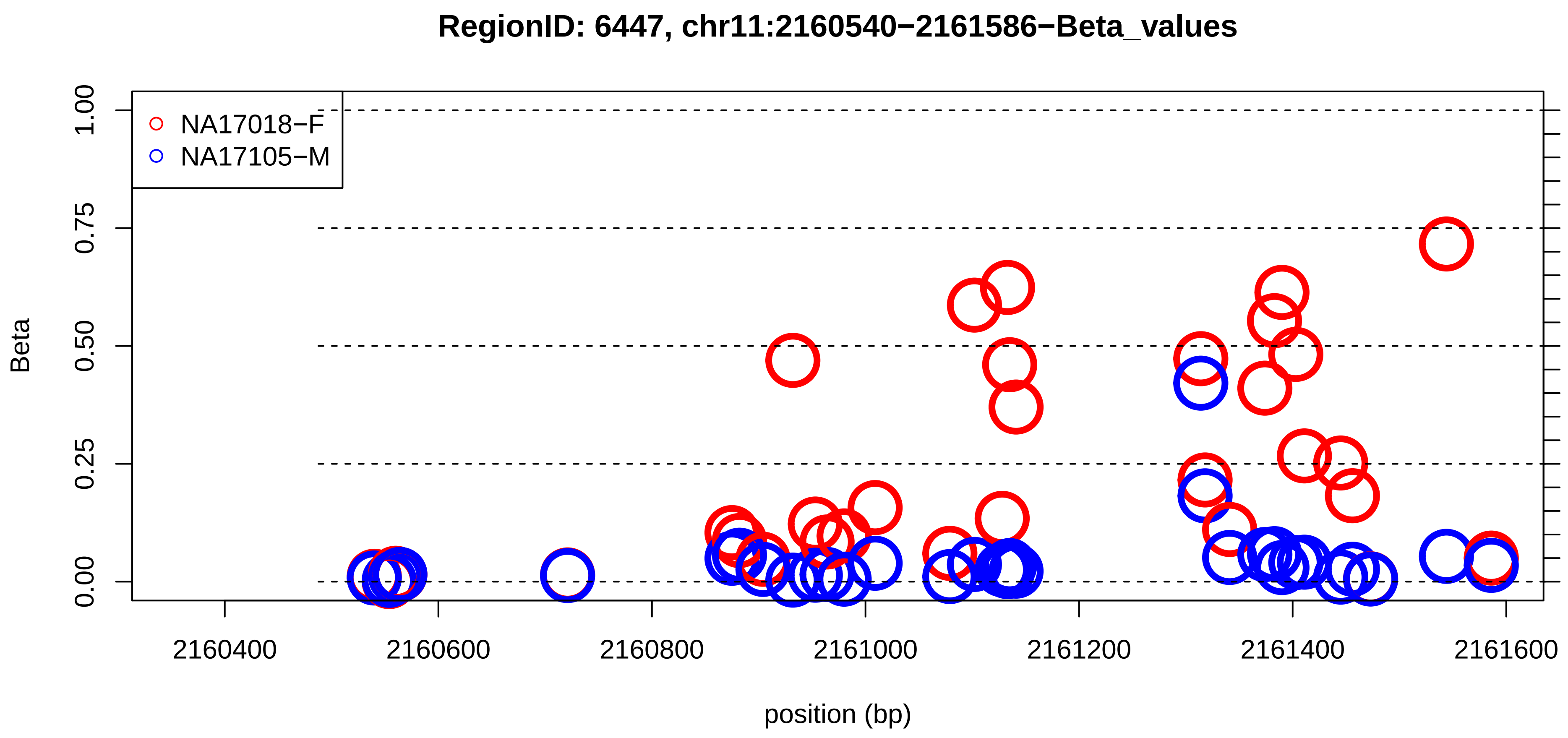

RegionID: 6447, chr11:2160540−2161586−Beta_values
NA17018−F
NA17105−M
1.00
0.75
0.50
0.25
0.00
Beta
2160400
2160600
2160800
2161000
2161200
2161400
2161600
position (bp)

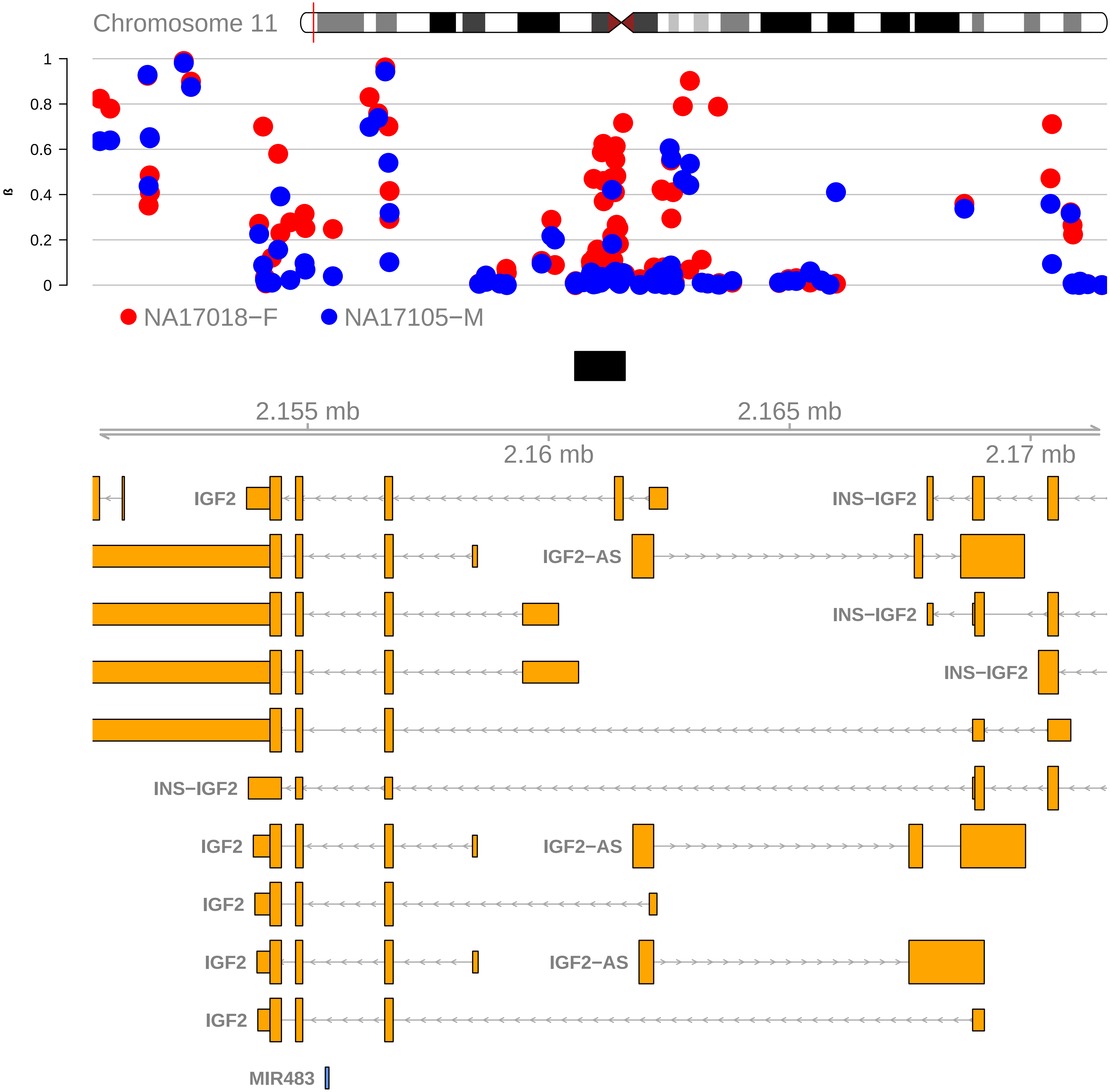

Chromosome 11
1
0.8
0.6
0.4
0.2
0
β
NA17018−F
NA17105−M
2.155 mb
2.16 mb
2.165 mb
2.17 mb
IGF2
INS−IGF2
IGF2−AS
INS−IGF2
INS−IGF2
INS−IGF2
IGF2
IGF2−AS
IGF2
IGF2
IGF2−AS
IGF2
MIR483
